# Wild or Tame? In Search for the Genetic Origin of Wild Boar ( *Sus scrofa* ) in Sweden

**DOI:** 10.1002/ece3.73369

**Published:** 2026-04-03

**Authors:** Anna M. Johansson, Anne‐Marie Dalin, Elisabeth Jonas, Sofia Mikko, Anna Malmsten

**Affiliations:** ^1^ Department of Animal Biosciences Swedish University of Agricultural Sciences Uppsala Sweden; ^2^ Department of Clinical Sciences Swedish University of Agricultural Sciences Uppsala Sweden; ^3^ Institute of Animal Sciences, Animal Breeding, University of Bonn Bonn Germany; ^4^ Independent Scholar Vallentuna Sweden

**Keywords:** diversity, domestic pig, introgression, origin, SNP

## Abstract

The wild boar ( *Sus scrofa* ) has been a part of the Swedish fauna for thousands of years. Although it became extinct in the 17th century, populations reemerged in the 1970s and 1980s after escapes from enclosures. The country of origin of the wild boars kept in these enclosures is unknown. This study aims to investigate the origin and level of genetic diversity of Swedish wild boars, as well as to compare them with Swedish domestic pigs. Swedish wild boar samples were collected from four regions of Sweden: Skåne, Blekinge, Södermanland and Uppland. In total, 107 wild boars and 427 domestic pigs were genotyped using the 80 K SNP chip. Wild boars from Skåne and Blekinge were shown to belong to the same genetic group, whereas the Södermanland and Uppland formed separate clusters. Results from both principal component analysis (PCA) and fixation index (F_ST_) analyses demonstrate that Swedish wild boars are genetically distinct from Swedish domestic pigs. Moreover, the ADMIXTURE analysis revealed no evidence of introgression from domestic pigs into Swedish wild boar populations. The genetic differentiation observed among Swedish wild boar populations further indicates that they originate from more than one external source population.

## Graphical abstract

Swedish wild boar samples were collected in four different regions of Sweden, Skåne, Blekinge, Södermanland and Uppland and genotyped using the 80 K SNP chip. Swedish wild boars are genetically distinct from Swedish domestic pigs. Wild boars from different Swedish regions are similar to different European population, which indicates that the Swedish wild boar populations originate from more than one external population.

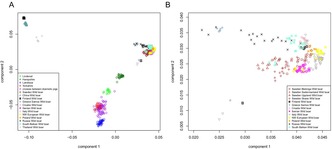

## 1 Introduction

The wild boar *(Sus scrofa)*, belonging to the family Suidae, is an ancestor of the domestic pig. Wild boars have a wide geographical distribution and inhabit most regions of Europe, southern Asia, and northern Africa. Domestication of wild boars occurred independently at two geographically distant locations, in East Asia and in the region of present‐day Türkiye (Giuffra et al. 2000; Kijas and Andersson 2001, Larson et al. 2005), resulting in clear genetic differentiation between European and Asian domestic pigs. As domestic pigs spread into Europe, extensive introgressions from local wild boar populations took place (Franz et al. 2019). In the 17th century, pigs from China were imported into the UK and the genomic footprint of this introgression can still be seen in the Yorkshire breed (also known as Large White) (Bosse et al. 2014) as well as in the mitochondrial DNA (mtDNA) of several Euro‐American pig breeds (Okumura et al. 2001).

Free‐ranging wild boars have been a part of the Swedish fauna for thousands of years. The species became extinct in the 17th century (Markström and Nyman 2002). The present population of free‐ranging wild boar in Sweden derives from an unknown number of captive wild boars that escaped from enclosures during the 1970s and 1980s. In addition, human activities, such as translocations and releases of animals into areas where wild boars were previously absent, may have influenced the dispersal and distribution of the species in Sweden. The official management strategy following the escapes was aimed at culling all wild boars found outside enclosures; however, these efforts were unsuccessful. By the mid‐1980s, several wild boar populations had become established in different parts of the country. In 1987, the Swedish parliament decided that the wild boar should once again be part of the Swedish fauna (Naturvårdsverket 2010), and today, the population is estimated to be 325,000 individuals by the Swedish Environmental Protection Agency (Naturvårdsverket 2025). The origin of the wild boars held in enclosures remains unknown. There are speculations and unconfirmed reports that wild boars were imported from different countries, as well as instances of crossbreeding with domestic pigs. However, neither the counties of origin nor any introgression from domestic pigs has ever been verified.

**FIGURE 1:**
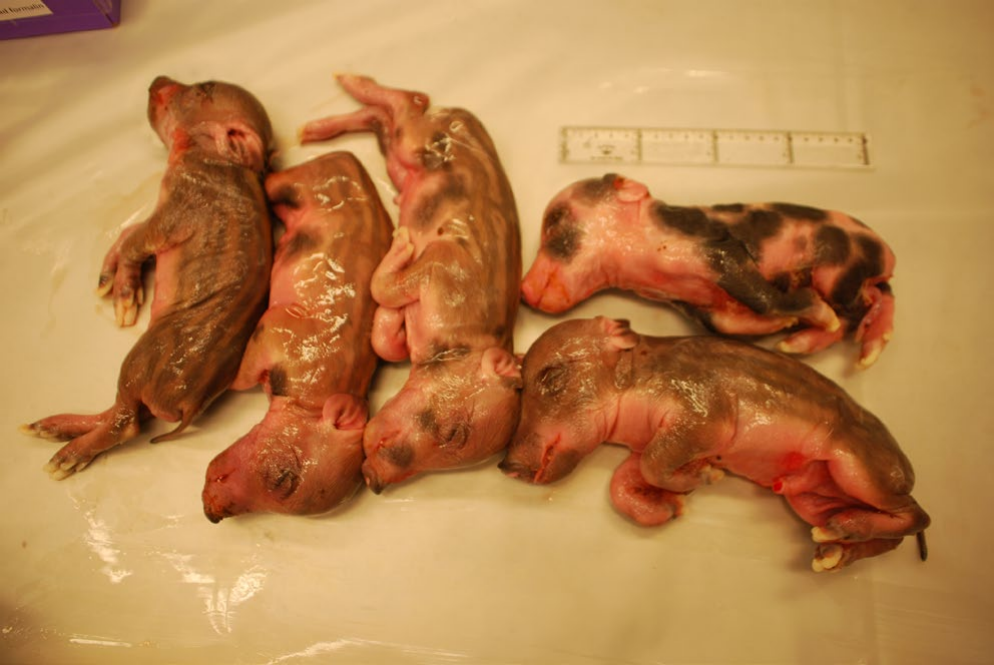
Litter of wild boar foetuses showing different coat colour phenotypes, suggesting introgression of domestic pig.

The genetic background of wild boar populations, and any potential influence of domestic pigs, is known to affect the litter size and growth rate (Booth 1995) as well as reproductive success (Fabbri et al. 2023). Bergman et al. (2013) analysed a part of the *MBL1* gene and identified an allele at a frequency of 35% in Swedish wild boars that may indicate interbreeding between wild boar and domestic pigs. In addition, variation in coat colour phenotypes among wild boars (Figure 1) have raised questions among hunters as to whether hybridisation between wild boars and domestic pigs occurred in the past. Nevertheless, further studies are required to determine the occurrence of genetic introgression from domestic pigs into the Swedish wild boar population.

The genetic diversity of wild boars, as well as possible introgression of domestic pigs into wild boar populations, has previously been studied using a range of molecular approaches. Microsatellite markers were used to investigate introgression from domestic pigs into wild boar populations (Frantz et al. 2013; Canu et al. 2014; Šprem et al. 2014). Genetic variation in mitochondrial DNA (mtDNA) has been used in several studies to infer the maternal origin of wild boars and domestic pigs in Europe (Okumura et al. 2001; Alves et al. 2010; Frantz et al. 2013; Kusza et al. 2014; Soria‐Boix et al. 2017). In addition, SNP‐chips, designed using information from domestic pigs, have been applied to study genetic diversity in wild boar populations (Goedbloed, van Hooft, et al. 2013; Goedbloed, Megens, et al. 2013; Herrero‐Medrano et al. 2013).

The objectives of this study were to investigate the genetic origin and diversity of wild boars in Sweden. Specifically, the study focused on three main aims. (1) to examine the genetic relationships within and among wild boar populations from different geographical locations in Sweden. (2) to compare Swedish wild boars with Swedish domestic pigs in order to identify the occurrence and potential sources of genetic introgression from domestic pigs into Swedish wild boar populations; and (3) to compare the genetic composition of Swedish wild boars with that of wild boar populations from other parts of Europe.

## 2 Materials and Methods

**FIGURE 2:**
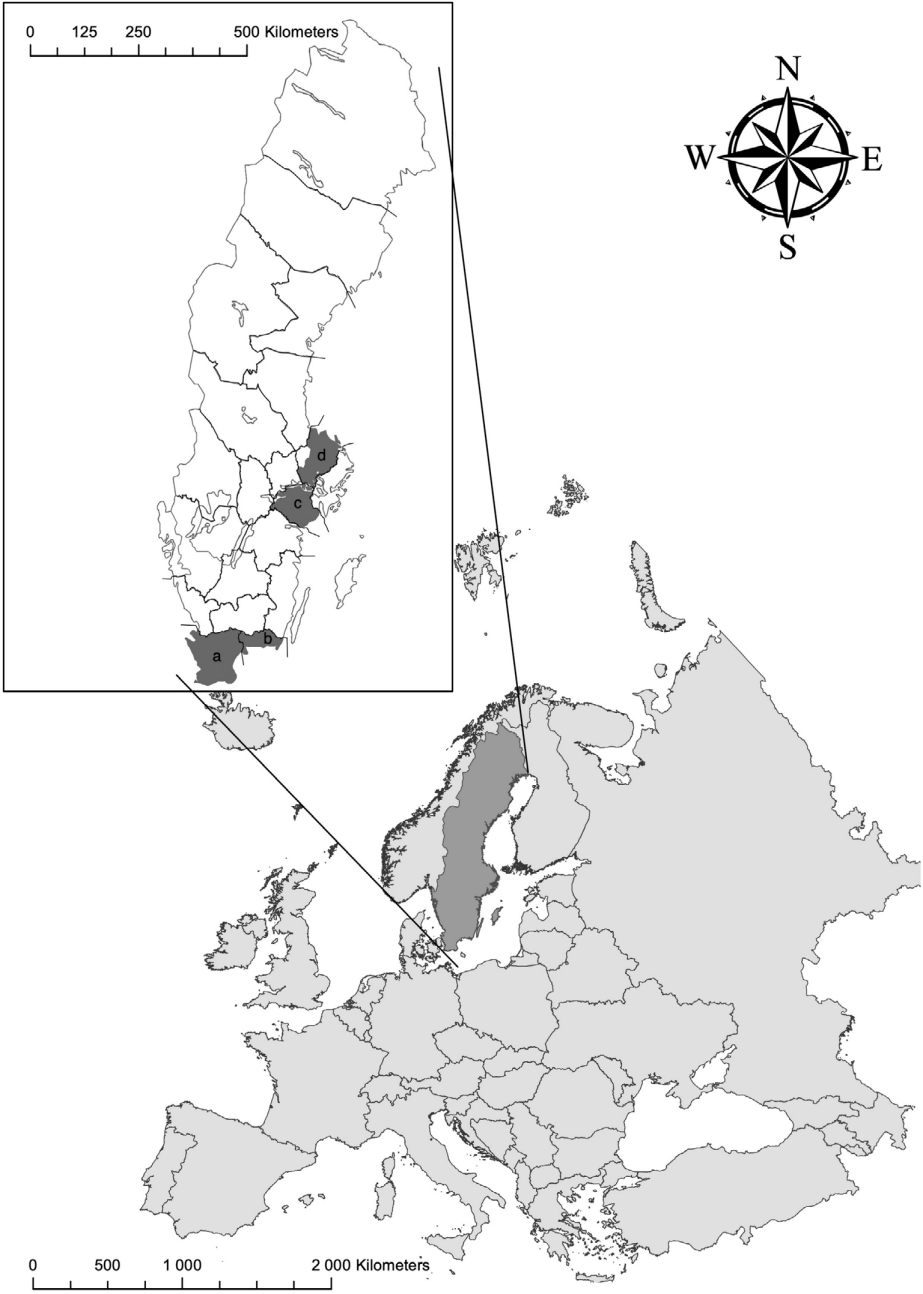
Map of Sweden highlighting the regions where wild boars were sampled. (a) Skåne, (b) Blekinge, (c) Södermanland, (d) Uppland.

Samples and information from wild boars were collected between January 2013 and December 2014 as part of a larger study on female wild boar reproduction, in which the samples were phenotypically described (Malmsten et al. 2017). Sampling was conducted during regular hunting at four hunting estates located in four Swedish regions (Skåne, Blekinge, Södermanland, and Uppland) in southern Sweden (Figure 2). Spleen or ear samples were collected from 107 hunted wild boars (Table 1) and stored in plastic bags at −20°C.

**TABLE 1:** Number of wild boar sampled in different Swedish regions and measures of genetic diversity.

Location	Number of ear samples	Number of spleen samples	Total number of samples	Number of samples after quality control	Mean observed heterozygosity (H_O_)	Mean expected heterozygosity (H_E_)	Inbreeding coefficient (F)
Blekinge	0	22	22	13	0.289	0.316	0.084
Skåne	0	30	30	10	0.277	0.316	0.122
Södermanland	5	20	25	13	0.291	0.316	0.077
Uppland	7	24	31	14	0.273	0.316	0.135
Total	12	96	107	50	0.283	0.316	0.104

Blood samples from Swedish Yorkshire (also known as Large White, *N* = 142), Hampshire (*N* = 24), Yorkshire crossed with other breeds (here referred to as Yorkshire cross; *N* = 19), Landrace (*N* = 40), Yorkshire × Landrace crosses (*N* = 39), and crossbreds of unclear composition (*N* = 13) were collected between 2011 and 2014. These samples were obtained for routine testing at the Animal Genetics Laboratory, Department for Animal Breeding and Genetics, Swedish University of Agricultural Sciences (SLU), as well as from different research projects, and were stored at the SLU Biobank in Uppsala. Some of these pigs were part of the Nordic Genetics breeding scheme, whereas others were collected from commercial farms in Sweden or from the Swedish Livestock Research Centre Lövsta, SLU (Jonas and Rydhmer 2018).

Samples from the Swedish breed Linderöd pigs (*N* = 43) were collected between 2019 and 2020, either as nasal swabs (PG‐100 from PERFORMAgene) by pig owners or from frozen meat samples obtained from slaughtered pigs. Total genomic DNA was extracted from the blood samples using the QIAsymphony DSP DNA Mini Kit (Qiagen, Hilden, Germany) according to the manufacturer's “High Content Protocol”, from the nasal swabs following the manufacturer's instructions for PERFORMAgene reagent (DNA Genotek Inc., Ottawa, ON, Canada), and from meat samples using the Qiagen Blood and Tissue Kit (Qiagen, Hilden, Germany). An overview of the domestic pig samples is provided in Table 2.

**TABLE 2:** Overview of samples genotyped in this study and the proportions that passed quality control and were polymorphic on the SNP array. F is inbreeding coefficient. H_O_ is mean observed heterozygosity. H_E_ is mean expected heterozygosity.

Population	Number of samples before quality control	Number of samples after quality control	Proportion of samples that passed quality control	Proportion of polymorphic markers (after quality control)	H_O_	H_E_	F
Wild boar	107	50	47%	79%	0.283	0.316	0.104
Linderöd	43	13	30%	76%	0.362	0.343	−0.056
Hampshire	24	24	100%	86%	0.316	0.314	−0.008
Landrace	40	40	100%	87%	0.328	0.320	−0.027
Yorkshire^a^	142	134	94%	92%	0.356	0.351	−0.013
Yorkshire cross	19	15	79%	91%	0.419	0.381	−0.098
Yorkshire × landrace	39	38	97%	94%	0.420	0.391	−0.074
Undefined crosses	13	8	62%	89%	0.398	0.378	−0.052
Total	427	322	75%	95%			

^a^ In the PCA analysis only 40 of these were used to get more even sample sizes of the different breeds and thereby avoid a bias in the PCA analysis.

Genotyping was performed using the GeneSeek Genomic Profiler Porcine 80 K SNP‐chip, which is based on the Illumina technology and contains 68,528 SNPs. Wild boar genotypes from the study of Yang et al. (2017) were added to our dataset for comparisons with wild boar populations from other countries.

Ethical permission was not needed for the collection of samples represented in the study, given that sample collection was the result of typical hunter harvest (i.e., wild boar in Sweden) or from production animals (breeds included in genotyping) in which sample collection did not deviate from standard handling practices for production animals or were collected post‐ mortem from animals slaughtered for consumption.

Custom‐made R scripts (R Core Team 2024) were used to combine the genotypes, remove duplicate samples, and create input files in PLINK format. Samples with a genotyping rate of at least 90% were retained, and markers were required to have a genotyping rate of at least 95%. Owing to the diversity of sample origins, and to ensure that minor alleles potentially present in only one breed were not discarded, no filtering on the basis of minor allele frequency was applied. In addition, deviations from the Hardy–Weinberg equilibrium were not considered, because of indications of high levels of inbreeding in some populations. By omitting these filters, the signals of inbreeding were preserved. PLINK 1.9 (www.cog‐genomics.org/plink/1.9/) was used for quality control and for most genetic analyses, whereas pairwise F_ST_ analyses were conducted using PLINK 2.0 (www.cog‐genomics.org/plink/2.0/) (Chang et al. 2015).

Of the 427 Swedish wild boar and domestic pig samples, 322 passed the quality control, and a total of 61,167 SNPs remained in the combined dataset. The same quality control parameters were applied to a separate dataset containing only Swedish wild boars, from which 50 samples and 57,282 SNPs passed quality control. An alternative quality control procedure was also tested, in which markers with low genotyping rates were removed prior to excluding samples with low call rates. However, this approach resulted in the removal of more than two‐thirds of the markers and still required the exclusion of 45 samples. This outcome indicated substantial issues with DNA quality in a subset of samples and supported the appropriateness of the quality control procedure applied in this study, which excluded a relatively large number of samples.

Heterozygosity was calculated using the “het” command in PLINK 1.9, and principal component analysis (PCA) was performed using the “pca” command in PLINK 1.9. For the PCA of the combined dataset, only 40 Yorkshire pigs were included to ensure comparable sample sizes across breeds and to avoid bias in the PCA result. The first and second principal components were plotted in R to visualise genetic differences among the populations.

To estimate the proportion of polymorphic markers in wild boar and domestic pig breeds, separate analyses were conducted for each population using the “freq” command in PLINK 1.9. The proportion of polymorphic markers with minor allele frequency larger than zero was then calculated. Population structure was inferred using ADMIXTURE version 1.3.0 (Alexander et al. 2009). Linkage disequilibrium pruning was performed using the parameters—indep‐pairwise 50 5 0.2, resulting in 12,876 remaining variants. ADMIXTURE analyses were conducted for *K* values between 2 and 25. The lowest cross‐validation error Data S2 was observed at *K* = 21, which was therefore selected as the optimal number of clusters, and results for *K* = 21 were plotted using a custom R script.

Overall F_ST_ was calculated in PLINK 1.9 using the “fst” command. Pairwise F_ST_ values were calculated in PLINK 2.0 using the “Weir‐Cockerham method” with breed names specified as phenotypes. A phylogenetic tree was constructed from the pairwise F_ST_ distance matrix using the BioNJ method implemented in Phylogeny.fr (Dereeper et al. 2008), and the resulting tree was visualised using TreeDyn at Phylogeny.fr.

## 3 Results

The proportion of samples that passed quality control was lower for wild boars and Linderöd pigs than for the other domestic pig breeds in this study (Table 2). Nevertheless, a total of 50 wild boar samples, representing all four Swedish regions, passed quality control (Table 1). The proportion of polymorphic markers was lowest in the Linderöd breed (76%) and second lowest in the Swedish wild boars (79%), which is clearly lower than in the purebred commercial pig breeds, where 86%–92% of markers were polymorphic (Table 2).

**FIGURE 3:**
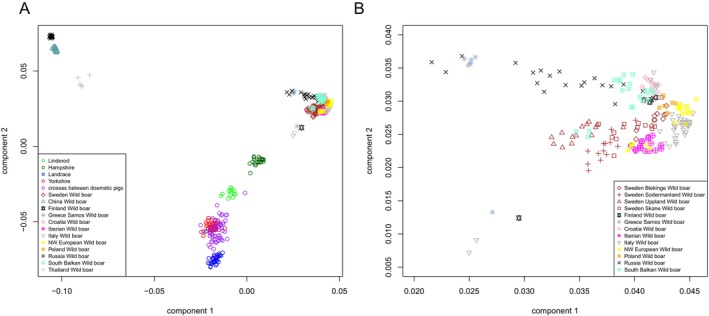
(a) A PCA of Swedish domestic pigs and wild boars, along with wild boars from several other countries, where multiple populations from a country are combined into one symbol. Also, the different types of Swedish crossbred pigs are combined. Only 40 Yorkshire individuals were included to ensure a sample size more comparable to the other populations. (b) A zoomed‐in view of part of Plot 3a, showing results only for the European wild boars, with wild boar individuals from different Swedish regions indicated by distinct symbols.

The PCA, including all populations, showed a clear separation between wild boars and all purebred Swedish domestic pigs (Figure 3a). The first principal component explains 46.0% of the total variation, whereas the second component explains 32.8%. Both components separated wild boars from the domestic pigs, indicating no recent admixture between wild and domestic pigs.

The Linderöd and Hampshire breeds formed distinct clusters, with all individuals from each population clustering together. A few Landrace, Yorkshire, and crossbred individuals clustered with the purebred and crossbred clusters, respectively. Among the domestic pig breeds, Hampshire was genetically closest to the European wild boar populations (Figure 3a). When focusing on wild boar populations, Swedish wild boars clustered partly with the wild boar populations from other regions of Europe. However, wild boars from Blekinge and Skåne were genetically closer to wild boars from Poland, whereas three individuals from the southern Balkan wild boar population clustered with wild boars from Uppland (Figure 3b).

**FIGURE 4:**
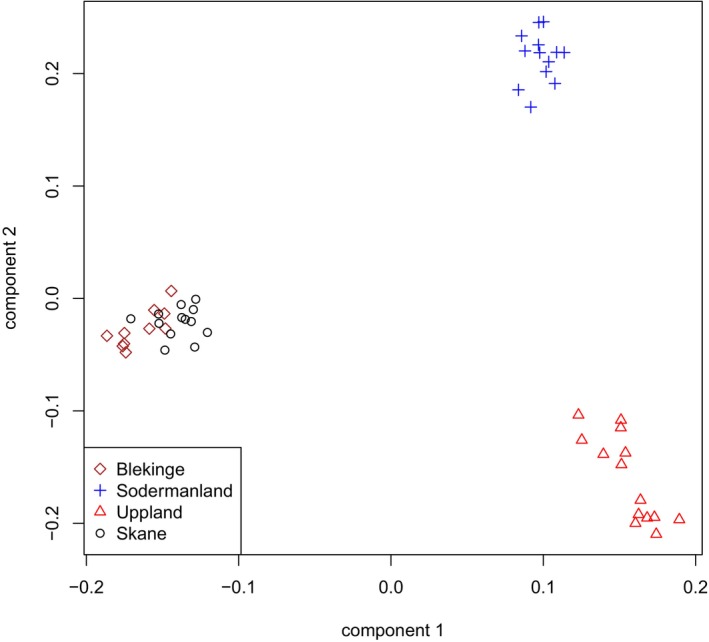
PCA of only Swedish wild boars showing three distinct clusters, with individuals from Blekinge and Skåne grouping together in the same cluster.

In the PCA including only Swedish wild boars, three distinct genetic groups were observed (Figure 4). Samples from Skåne and Blekinge cluster together, whereas samples from Södermanland and Uppland each formed separate clusters. In this analysis, the first principal component explained 5.8% of the variation, and the second component explained 3.0%.

**FIGURE 5:**
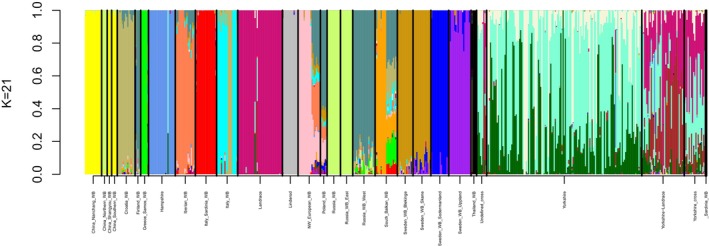
ADMIXTURE analysis with K = 21, showing wild boars from Skåne and Blekinge having the same ancestry and the Uppland and Södermanland populations having distinct ancestries.

Results from the ADMIXTURE analysis at K = 21 are shown in Figure 5. Swedish wild boars from the southernmost regions (Skåne and Blekinge) displayed highly similar ancestry profiles, aligning with the PCA results. Wild boars from the regions surrounding Stockholm (Södermanland and Uppland) were genetically distinct from each other and from the southern populations. A small number of individuals showed minor ancestry contributions from other Swedish wild boar populations, and in a few cases, Swedish wild boars shared very small ancestry proportions with wild boars from other European populations.

The ancestry component dominating wild boars from Skåne and Blekinge is also present, at lower proportions, in wild boars from Poland and in a small number of individuals from north‐western Europe. The ancestry dominating wild boars from Södermanland was also present at low proportions in wild boars from Poland and at very low levels in individuals from several other wild boar populations, as well as in a few domestic pigs. Overall, no clear shared ancestry between Swedish wild boars and Swedish domestic pig populations was detected in any of the four regions. However, some wild boars from Skåne appeared to share a small proportion of ancestry with Landrace pigs. In addition, several samples supposed to be purebred domestic pigs appeared instead to be crossbred according to the ADMIXTURE results, consistent with their placement outside breed‐specific clusters in the PCA. With the exception of one Linderöd pig showing a small proportion of shared ancestry with wild boars from Uppland and Södermanland, Swedish domestic pigs did not share ancestry with wild boars.

**FIGURE 6:**
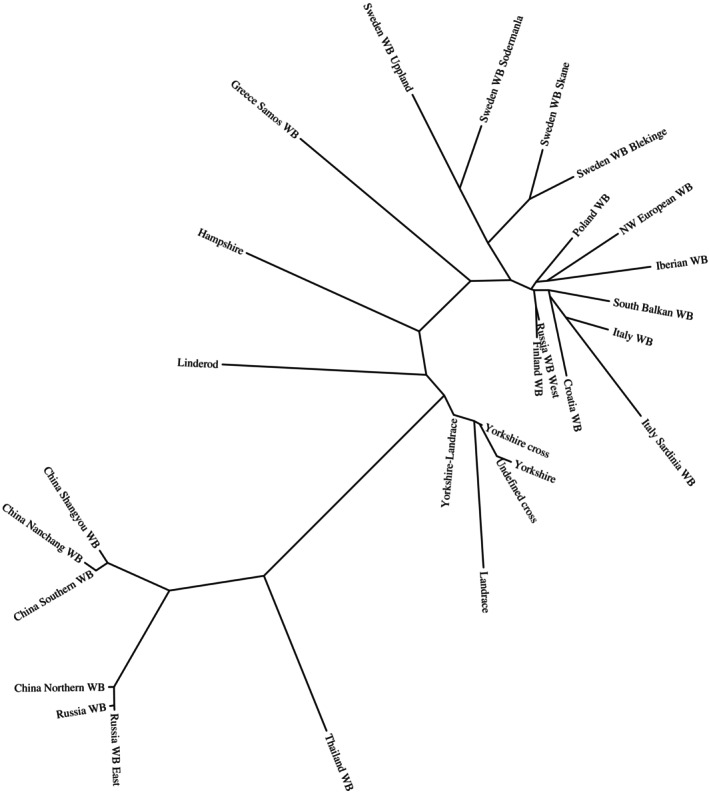
Phylogenetic tree on the basis of a distance matrix with the pairwise F_ST_ values shows that the Swedish wild boars are closer to other European wild boar populations than to domestic pigs and to Asian wild boar populations.

The overall F_ST_ value for our combined dataset, including wild boar samples from Yang et al. (2017) was 0.29 (Data S1). Further, only results involving Swedish populations are presented. Swedish wild boars showed the highest F_ST_ values when compared with wild boar populations from Russia, China, and Thailand, with values exceeding 0.50. The lowest F_ST_ was observed between the Swedish wild boar populations from Blekinge and Skåne (0.07), followed by the comparison between Uppland and Södermanland (0.12). The highest F_ST_ value among Swedish wild boar populations was observed between Blekinge and Uppland (0.20). Wild boars from Blekinge and Skåne showed lower F_ST_ values in comparisons with wild boars from Finland and western Russia than in comparisons with wild boars from Uppland or Södermanland. The F_ST_ values between Skåne or Blekinge and Poland were within the same range as those observed between Skåne or Blekinge and Södermanland or Uppland. All investigated Swedish wild boar populations showed substantially lower F_ST_ values in comparison with European wild boar populations than in comparison with Swedish domestic pig breeds. The phylogenetic tree on the basis of pairwise F_ST_ values clearly illustrates that Swedish wild boars are more closely related to other European wild boar populations than to Swedish domestic pigs or Asian wild boars (Figure 6).

Mean observed heterozygosity in the four Swedish wild boar populations ranged from 0.273 to 0.291, with an overall mean of 0.283 across all wild boar samples (Table 1). The observed heterozygosity was, on average, lowest in Uppland (0.273) and highest in Södermanland (0.291), indicating that genetic diversity was highest in the Södermanland population. In all four regions, observed heterozygosity was lower than the expected heterozygosity (0.316), indicating inbreeding in Swedish wild boar populations. The individual inbreeding coefficient, calculated from the difference between observed and expected heterozygosity, ranged from −0.038 to 0.274, with a mean value of 0.104. Wild boars from Uppland and Blekinge showed, on average, higher levels of inbreeding than those from Skåne and Södermanland. Observed heterozygosity in Swedish wild boar populations was lower than in all Swedish domestic pig populations included in this study (Table 2). Expected heterozygosity in Swedish wild boar populations was similar to that observed in the domestic pig breeds Landrace and Hampshire, but lower than in Yorkshire and Linderöd pigs. As expected, crossbred pigs showed higher mean heterozygosity than purebred pig breeds. Overall, inbreeding levels were clearly higher in Swedish wild boars than in Swedish domestic pigs (Table 2). Observed heterozygosity in Swedish wild boar populations was also lower than in most wild boar populations from Yang et al. (2017) that are reported in Data S3.

## 4 Discussion

To our knowledge, this is the first study of genome‐wide genotypes from Swedish wild boar populations and the first attempt to shed light on the origin of four of the current wild boar populations in Sweden.

In our study, a relatively large proportion of wild boar and Linderöd pig genotypes failed quality control. For the wild boar, this may be due to DNA degradation during sample shipment, and for the Linderöd pig, due to nasal swabs giving low quality of DNA, resulting in suboptimal SNP detection. A large proportion of the wild boar samples failed quality control in the study by Dadousis et al. (2022). The low proportion of polymorphic SNPs observed in the Linderöd pig is instead most likely a consequence of the severe bottleneck the breed experienced when it was close to extinction.

Most wild boar populations from the dataset of Yang et al. (2017) show a lower proportion of polymorphic markers than the Swedish wild boars; however, in many cases, this is likely due to small sample sizes in several populations (Data S3). When comparing the four Swedish wild boar populations, higher mean observed heterozygosity was observed in Skåne and Södermanland than in Blekinge and Uppland. The observed heterozygosity on the basis of SNP data for all four Swedish regions was lower than that reported for most wild boar populations in the dataset of Yang et al. (2017), with wild boars from Sardinia being the only population showing lower heterozygosity than the Swedish wild boars (Data S3). By contrast, observed heterozygosity in Swedish wild boars was higher than that reported for wild boars from the Netherlands and Germany by Goedbloed, Megens, et al. (2013). Swedish wild boars also exhibited higher observed heterozygosity than the Central and Continental group, but clearly lower values than those reported for wild boars from Samos in the study of wild boar in the Balkan study by Alexandri et al. (2017).

The Swedish heritage breed Linderöd is of particular interest in comparison with the Swedish wild boar, as these pigs are often kept outdoors, which increases the risk of crossbreeding with wild boar. It is therefore of interest not only to detect any admixture of Linderöd pigs into wild boar populations, but also the potential introgression of wild boar into Linderöd pigs, which has been speculated by some. The clear separation of Linderöd pigs and wild boar into distinct clusters in the PCA analyses indicates that no recent admixture has occurred. This conclusion is further supported by the admixture analysis. One Linderöd pig showed a small proportion of shared ancestry with wild boars from Uppland and Södermanland, indicating wild boar ancestry several generations ago. None of the wild boar samples in our study showed any Linderöd pig ancestry. In addition, the F_ST_ values between Linderöd pigs and all Swedish wild boar populations were relatively high (ranging from 0.339 to 0.359), further supporting the absence of recent admixture between these populations. Moreover, F_ST_ values between Swedish wild boar populations and other Swedish domestic pig breeds were higher than those observed between Swedish wild boars and other European wild boar populations, showing that Swedish wild boars are more closely related to other European wild boar populations than to Swedish domestic pigs. Since wild boars were absent from Sweden for a few 100 years, there have been fewer opportunities for admixture than in countries where wild boars have been continuously present. There are other methods of detecting admixture that are not explored within the scope of this study, and some methods could potentially give different results. This is something that may be further explored in future studies.

The fact that the samples from Skåne and Blekinge cluster together is not surprising, as these regions are geographically close to each other in the southernmost part of Sweden (Figure 2). Although they cluster closely together in the PCA plot, they exhibit different levels of heterozygosity. This could be explained by the fact that animals in the area in Skåne descend from animals in Blekinge and have gone through bottlenecks that have decreased the heterozygosity. The F_ST_ analysis supports the conclusion from the PCA, with Swedish wild boars from Skåne and Blekinge showing lower pairwise F_ST_ values than those observed in other pairwise comparisons among Swedish wild boar populations. The admixture analysis also confirms the close genetic connectedness between these two populations in southern Sweden.

The comparison of Swedish wild boar with wild boar populations from other countries in the PCA analysis showed that Swedish wild boars from Uppland clustered closely with some wild boars from the southern Balkans, whereas some Swedish wild boars from Skåne and Blekinge clustered near wild boars from Poland.

In addition, some wild boars from Södermanland, Skåne, and Blekinge were relatively close to wild boars from north‐western Europe, Italy, and the Iberian Peninsula, and one wild boar from Skåne clustered with wild boars from Croatia, Finland, and western Russia. Furthermore, wild boars from southern Sweden (Skåne and Blekinge) displayed lower F_ST_ values in comparison with wild boars from western Russia and Finland than in comparisons with other Swedish populations. The ADMIXTURE analysis also indicated some shared ancestry between Swedish wild boars from Skåne and Blekinge and wild boars from Poland, in agreement with the PCA analysis. However, the ADMIXTURE analysis did not reveal any shared ancestry between wild boars from the southern Balkans and the Swedish wild boar population from Uppland. Taken together, the results from all analyses indicate that Swedish wild boar populations are divided into three genetic groups and are more closely related to other European wild boar populations than to Swedish domestic pigs or Asian wild boars.

Overall, these results indicate that wild boars have entered Sweden from more than one European source population. Given that the current wild boar populations in Sweden are only a few decades old, the pronounced genetic differences observed between the three genetic groups would probably be unlikely to develop in such a short time if they had all originated from a single source population. It is therefore likely that wild boars were imported into enclosures in Sweden from different countries at different times. No clear evidence of introgression from Swedish domestic pig breeds was detected in this study. However, the indications of domestic pig introgression into Swedish wild boars reported on the basis of the *MBL1* gene (Bergman et al. 2013) and on coat colour variation may instead reflect introgression events that occurred prior to the importation of wild boars into Swedish enclosures. Genes responsible for coat colour variation may have been introgressed from domestic pigs in other countries many generations ago. If only a small proportion of domestic pig DNA is present in the Swedish wild boar population, it would be difficult to detect. Furthermore, if we do not have samples from the as‐yet unknown pig breed(s) that contributed to the genetic makeup of Swedish wild boars it will not be possible to identify genetic similarities between these breeds and Swedish wild boars.

## Author Contributions

**Anna M. Johansson:** formal analysis (equal), methodology (equal), visualization (equal), writing – original draft (equal), writing – review and editing (equal). **Anne‐Marie Dalin:** conceptualization (equal), resources (equal), writing – review and editing (equal). **Elisabeth Jonas:** resources (equal), writing – review and editing (equal). **Sofia Mikko:** conceptualization (equal), methodology (equal), supervision (equal), writing – review and editing (equal). **Anna Malmsten:** conceptualization (equal), methodology (equal), resources (equal), writing – review and editing (equal).

## Funding

The Swedish Environmental Protection Agency and the Swedish Association for Hunting and Wildlife Management supported the collection of samples from wild boar. Genotyping of the Swedish domestic pigs was partly funded by Formas (2013‐00312_Formas) and a Postdoc‐Fund from the Carl Tryggers Stiftelse.

## Conflicts of Interest

The authors declare no conflicts of interest.

## Supporting information


**Data S1:** Supporting Information.

**Data S2:** Supporting Information.

**Data S3:** Supporting Information.

## Data Availability

The genotype data are available as files in PLINK format that can be downloaded from dryad https://doi.org/10.5061/dryad.xwdbrv1sg.
