# Supplementary material for: Wild or Tame? In Search for the Genetic Origin of Wild Boar ( Sus scrofa ) in Sweden
Source: Ecol Evol. 2026 Apr 3;16(4):e73369. doi: 10.1002/ece3.73369 (PMC13052102; doi:10.1002/ece3.73369)
Supplement: Supplementary file 2 — Data S2: Supporting Information. [file ECE3-16-e73369-s001.pdf]

K	CV error
2	0.69163
3	0.63791
4	0.60607
5	0.58913
6	0.57461
7	0.56810
8	0.56161
9	0.55501
10	0.55266
11	0.54659
12	0.54505
13	0.54381
14	0.53888
15	0.53674
16	0.53560
17	0.53412
18	0.53276
19	0.52984
20	0.53017
21	0.52763
22	0.53128
23	0.52915
24	0.52920
25	0.53011
