# Supplementary material for: Wild or Tame? In Search for the Genetic Origin of Wild Boar ( Sus scrofa ) in Sweden
Source: Ecol Evol. 2026 Apr 3;16(4):e73369. doi: 10.1002/ece3.73369 (PMC13052102; doi:10.1002/ece3.73369)
Supplement: Supplementary file 3 — Data S3: Supporting Information. [file ECE3-16-e73369-s003.pdf]

Additional File 3

Overview of diversity in wild boar samples from Yang et al, using SNP data that overlaps with the Swedish SNP data

Population	Number of samples	Proportion of polymorphic markers	Mean observed heterozygosity (H_O)	Mean expected heterozygosity (H_E)	Inbreeding coefficient (F)
China Nanchang	15	0.640	0.332	0.334	0.005
China Northern	5	0.467	0.390	0.359	-0.086
China Shangyou	4	0.515	0.427	0.372	-0.147
China Southern	5	0.563	0.380	0.361	-0.053
Croatia	16	0.666	0.339	0.341	0.006
Greece Samos	7	0.583	0.301	0.318	0.053
Iberian	18	0.684	0.312	0.344	0.093
Finland	5	0.654	0.347	0.363	0.044
Italy Sardinia	20	0.749	0.256	0.294	0.131
Italy	19	0.695	0.293	0.337	0.130
NW European	20	0.694	0.304	0.330	0.079
Poland	6	0.611	0.378	0.362	-0.045
Russia	12	0.547	0.333	0.331	-0.008
Russia East	11	0.537	0.345	0.334	-0.034
Russia West	20	0.844	0.314	0.319	0.014
South Balkan	20	0.740	0.301	0.331	0.091
Thailand	5	0.549	0.428	0.366	-0.170
